# Serological and molecular detection of infectious laryngotracheitis virus in chickens in Central Gondar Zone, Ethiopia

**DOI:** 10.3389/fvets.2025.1517373

**Published:** 2025-03-03

**Authors:** Omima Adam, Omolade A. Oladele, Tadesse Mihret Yimam, Belayneh Getachew, Getaw Deresse, Kenaw Birhanu, Abinet Legesse, Takele Abayneh Tefera, Molalegne Bitew

**Affiliations:** ^1^Animal Resource Research Corporation, Khartoum, Sudan; ^2^Avian Disease Unit, Department of Veterinary Medicine, University of Ibadan, Ibadan, Nigeria; ^3^University of Gondar, Gondar, Ethiopia; ^4^National Veterinary Institute, Debre Zeit, Ethiopia; ^5^Health Biotechnology Directorate, Bio and Emerging Technology Institute, Addis Ababa, Ethiopia

**Keywords:** chicken, ILT virus, ICP4 gene, ELISA, PCR, isolation, seroprevalence, Amhara

## Abstract

**Introduction:**

Infectious laryngotracheitis (ILT) is a highly contagious upper respiratory tract disease of chickens caused by a Gallid herpesvirus 1 (GaHV-1). The current study was to establish molecular evidence of Infectious laryngotracheitis virus (ILTV) in the Amhara region, Ethiopia, and determine its seroprevalence in areas of high chicken population and assess the risk factors associated with the disease.

**Methods:**

Serological study was conducted on 385 serum samples collected from commercial and backyard chickens in the study area, and the presence of antibodies against ILTV was determined by indirect ELISA. In addition, oropharyngeal swab samples were collected from chickens suspected of ILT infection and inoculated into embryonated chicken eggs through the Chorioallantoic membrane (CAM) route for isolation of the virus. Isolates were confirmed using polymerase chain reaction (PCR) upon amplification of ICP4 gene. Furthermore, potential factors were recorded, and their association with the virus seropositivity assessed.

**Results:**

The overall seroprevalence of ILT in the study area was 19.4%. A significant difference (*P* < 0.05) among districts, and between commercial (14.2%) and backyard (22.9%) production systems was observed (*P* < 0.05). Significantly higher seroprevalence was observed in layers compared to broilers and dual-purpose chickens however, there were no significant differences in prevalence based on age and sex. Of all (*n* = 27) tested oropharyngeal swab samples, four were positive for ILTV by PCR targeting a 688 bp region of ICP4 gene. Three of the PCR positive cases were from backyard chickens, while one was from commercial chicken farms. Based on oropharyngeal samples tested using PCR, a quarter of the samples were positive for ILT.

**Discussion:**

The result confirms the presence of ILT infection in the Amhara region of Ethiopia using serological and molecular methods. The study shows chickens shed the virus potentially spreading the infection to other birds. Vaccination strategy, strict biosecurity measures, rapid diagnosis, and detection of latent carriers are recommended to control and eradicate the disease. Further studies on clinical cases and the molecular characterization of the target gene are needed to identify circulating strains.

## 1 Introduction

Infectious laryngotracheitis is a respiratory disease of chickens, which is found all over the world, caused by ILTV, also known as gallid herpesvirus 1(GaHV-1) (1), classified under the genus Iltovirus, in the subfamily Alphaherpesvirinae. It is a double-stranded DNA virus that is enveloped, non-segmented, and linear (2). May and Tittsler (41), were the first to characterize infectious laryngotracheitis as a distinct disease entity; they recorded an outbreak on a farm in Rhode Island in 1923. However, several of the early workers pointed out that the disease had most likely been present in North American chickens for some years before 1925 (3). Infectious laryngotracheitis is on the Office International des Epizooties' (OIE) List B. The virus is one of the most prevalent diseases seen among chickens with contagious respiratory infections (4).

Gasping, depression, nasal discharge, conjunctivitis, and the secretion of bloody mucus are major symptoms associated with the acute form of the disease in chickens (5). ILTV also causes coughing, decreased egg production in layers, and mortality (4). Postmortem in dead chickens show hemorrhages and mucus plugs in the trachea (6). The severity of clinical symptoms is determined by the virulence of a particular strain or isolate, with mortality rates ranging from 0% to 70% (7). The virus can establish lifelong latent infections in the central nervous system, particularly within the trigeminal ganglion, after a 6–12-day incubation period following natural infection by ocular or respiratory routes. Although difficult to detect, sporadic reactivations can result in productive virus replication in the respiratory system, leading to shedding and transmission to susceptible animals (1). Infectious laryngotracheitis virus mostly found in chickens, but it's also been shown in pheasants and turkeys that were experimentally infected (8).

Chickens can be infected through the upper respiratory tract and ocular routes (9). Sanitary barriers and hygienic procedures play a vital impact in the severity of viral diseases in afflicted farms (10). ILT's high contagiousness is owing to the virus's ease of transmission and propagation, which is aided by sick chickens fomites, lack of biosecurity measures, animal movement, and inappropriate disposal of contaminated litter (11). Transmission occurs commonly from acute cases. Clinically inapparent infection can persist for long periods with intermittent re-excretion of the virus, serving as a potential source of disease transmission. Studies have shown that recombination between two or more varying strains can produce highly virulent and transmissible agents (12). Vaccination using live attenuated or recombinant viral vector vaccines is the most effective way to prevent ILT infection (13). Vaccination with two types of ILTV live attenuated vaccines, the chicken embryo origin (CEO), which is attenuated by sequential passages in embryonated eggs, and the tissue-culture origin (TCO), which is generated by sequential passages in tissue culture, is commonly used. A study showed that, live attenuated vaccines, particularly the CEO, can spread from bird to bird in close proximity and is able to revert to virulence after a few passages (14).

ILTV is found throughout the world, and several epidemiological investigations to detect circulating ILTVs undertaken in various countries (9). The virus has been identified in several nations where intensive chicken production is practiced, including North America, South America, Europe, China, Southeast Asia, and Australia. Laboratory diagnosis is essential to distinguish ILT from other diseases with similar clinical signs and lesions such as infectious bronchitis, Newcastle disease, avian influenza, infectious coryza, and mycoplasmas (15).

Only a few studies on ILT have been undertaken in Ethiopia, with the majority of them taking place in commercial and backyard chicken flocks. Previous reports by (10) and (16) indicated a higher seroprevalence of ILTV in chickens (without detecting the virus) in South and Central Ethiopia (10, 16), and another recent study has for the first time confirmed the virus in diseased chickens in the open markets of Addis Ababa, two genes were sequenced and the analysis showed that vaccine-like strains of ILTV were circulating (17). Indicated that the diseases possibly cause a substantial productive loss in the country, and more studies using a combination of molecular and serological techniques are needed to cover more regions of the country, create awareness about the disease, and make recommendations on prevention and future research. Therefore, the objective of this study is to determine seroprevalence, detect ILTV using molecular methods, and assess risk factors associated with infectious laryngotracheitis in chicken production systems in Amhara Region, Ethiopia.

## 2 Materials and methods

### 2.1 Study area

This research was carried out in Central Gondar Zone in the Amhara National Regional State (ANRS), Ethiopia. The ANRS is one of the largest states in Ethiopia, holding a huge poultry population. Among the different administrative zones of the ANRS, the Central Gondar City is geographically located between coordinates 12.3°-13.38° N latitudes and 35.5°-38.3° E longitudes, and has an altitude ranging between 1,750 and 2,600 m above sea level. It has an average annual rainfall of 1047.6 mm, mean maximum temperature of 27.4°C, and mean minimum temperature of 14.7°C and relative humidity of 45% (7).

The study area is a remarkable source of livestock, which contribute to the country's GDP with about 737,713 sheep, 1,479,366 goats, 3,234,012 cattle, 392,546 donkeys, 12,252 mules 39,178 horses and 3,310,498, chickens are kept in this area (18). The samples were collected from four districts of Gondar city (Gondar, Maksegneit, West Dembya, and East Dembya).

### 2.2 Study design, sampling technique, and sample size determination

Commercial and backyard chickens in Amhara region, Ethiopia were sampled from February to July 2022 in a cross-sectional study design. Districts and commercial chicken farms were selected purposely. Households with backyard chicken and individual chickens for blood sampling were selected haphazardly mimicking random sampling. Clinically ill animals with signs of nasal discharge, dyspnea and coughing were selectively sampled for viral detection, and blood samples were obtained from apparently healthy chickens. It was possible to collect a total of 27 oropharyngeal swabs from diseased chickens. For the sero-epidemiology, 385 blood samples were collected using (19) formula taking an expected prevalence of 50% and absolute desired precision of 5% at 95% confidence level (19).

### 2.3 Sample collection

#### 2.3.1 Source of birds

The chickens in this study were of existing exotic (Bovance and Sasso) and local breeds (Backyards chickens). Specimens were collected from backyard and commercial flocks located in four different districts in Central Gondar zone, Ethiopia (Gondar, Maksegnit, West Dembya, and East Dembya) (Table 1). The examined flocks were at 1–13 months age whose purpose ranged widely from egg production and meat production to dual purpose, and all flocks were not vaccinated against ILTV. During samples collection, potential risk factors such as flock history (sex, age, type of production, and breed) were noted. The informed consent was obtained from owners of the chickens to collect the samples.

**Table 1:** Farms monitored for specific antibody to ILTV.

**Location**	**Farm ID**	**Type of Chicken**	**No of samples/Total population of farm**
East Dembya	Farm-1	Commercial	20/1,700
	Farm-2	Commercial	11/900
	Farm-3	Commercial	13/2,400
	Farm-4	Backyard	5/17
	Farm-5	Backyard	15/44
	Farm-6	Backyard	4/12
	Farm-7	Backyard	3/6
	Farm-8	Backyard	15/50
	Farm-9	Backyard	22/60
Gondar City	Farm-1	Commercial	12/2,500
	Farm-2	Commercial	11/1,000
	Farm-3	Commercial	16/3,000
	Farm-4	Backyard	20/30
	Farm-5	Backyard	5/15
	Farm-6	Backyard	15/25
	Farm-7	Backyard	20/35
	Farm-8	Backyard	12/22
	Farm-9	Backyard	8/11
Maksegnit	Farm-1	Commercial	22/1,600
	Farm-2	Commercial	15/1,000
	Farm-3	Commercial	27/2,300
	Farm-4	Backyard	5/10
	Farm-5	Backyard	8/13
	Farm-6	Backyard	10/20
	Farm-7	Backyard	11/22
West Dembya	Farm-1	Commercial	3/300
	Farm-2	Commercial	4/500
	Farm-3	Backyard	23/50
	Farm-4	Backyard	15/30
	Farm-5	Backyard	15/35
Total			385/17,707

#### 2.3.2 Blood sample collection

A total of 385 blood samples (2–3 mL) were collected from backyard and commercial chickens (Table 2) from the wing vein using 5 mL syringes and 21-gauge needles. The blood samples were kept at room temperature overnight to produce serum. Serum samples were decanted into cryovial tubes and transported using an icebox to the National Veterinary Institute (NVI), Ethiopia, where they were kept at −20°C.

**Table 2:** Number of blood samples from local and commercial breed.

**Breed**	**Name**	**No. of samples**	**Percent (%)**
Local	Tilili ecotype	144	37.40
Exotic	Bovance	104	27.01
	Sasso	137	35.58
Total		385	100

#### 2.3.3 Oropharyngeal swabs

Twenty-seven oropharyngeal swab samples were collected from backyard and commercial flocks (1–3 samples from each flock) in the study area which display nasal discharge, dyspnea and coughing. Sterile cotton-tipped swabs were introduced into the mouth of each bird and gently swabbing the oropharynx. The samples were placed directly into sterile labeled cryovial tubes containing a 1.5 mL virus transport medium and transported on ice to the Virology Laboratory, NVI, Ethiopia, where they were stored at −80°C. A total of 27 samples, preserved in sterile PBS Phosphate- Buffered Saline (PBS) containing antibiotics and antifungal drug, were collected. A 10% of sample suspension made using PBS was centrifuged at 4,500 rpm for 10 min and the supernatant was used for virus isolation and DNA extraction.

### 2.4 Serological analysis for infectious laryngotracheitis virus antibodies

Indirect Enzyme linked Immunosorbent Assay (ELISA) technique was carried out using commercial Indirect ELISA kit (iELISA) (IDvet Screen^®^ ILT Indirect, 310 rue Louis Pasteur, 34790 Grabels, France) to detect the specific antibodies for GaHV-1 following the instructions of the manufacturer. After the microtiter plate was placed in the ELISA reader, the intensity of the color produced by the reaction was measured photometrically at 450 nm wavelength, then the values of sample optical density (OD sample) and the values of positive control optical density (ODPC) were noted. The test was considered valid when the mean OD value of the positive control was greater than 0.250 and when the ratio of the mean values of the positive and negative control was greater than 3 (ODpc/ODnc > 3). Test positivity or negativity was based on the formula indicated below:


$$\frac{S}{P}=\frac{ODS-ODNC}{ODPC-ODNC}\;$$

Where S/P: sample to positive ratio, ODS: sample optical density, ODNC: optical density of the negative controls, ODPC: optical density of the positive controls. Sample to positive (S/P) ratios of ≤ 0.3 and > 0.3 were interpreted as negative and positive, respectively.

The result was considered positive when the sample S/P ratio is greater than 0.3 (Titer > 611 per microliter). If the S/P ratio of the sample is less than 0.3 (Titer < 611 per microliter) it was interpreted as negative.

### 2.5 Molecular detection of ILTV

#### 2.5.1 Isolation of ILTV in embryonated chicken eggs

The swab samples (*n* = 27) in 10% PBS (pH 7.2) solution was centrifuged at low speed to remove debris. Aliquots of 0.2 and 0.3 mL of the supernatant fluid was inoculated on the dropped Chorioallantoic membrane (CAM) of at least two, 9–11 day-old embryonated chicken eggs of specific pathogen free (SPF) chicken. The eggs were sealed with paraffin wax, incubated at 37°C and candled daily for 4–7 days. During incubation, eggs with dead embryos were chilled at 4°C. After the last day of incubation, eggs with live embryos were chilled at 4°C overnight. The CAMs were harvested and homogenized in PBS. Homogenized CAM samples were re-passaged three times in SPF embryos. (20) explained the difficult of isolation of low virulent ILTV, also (21) described the limited propagation of ILTV isolated from mild cases. Therefore, three consecutive passages were performed for ILTV negative samples.

#### 2.5.2 Extraction of viral nucleic acid

As stated earlier, swab samples (*n* = 27) were used for DNA extraction and virus isolation. DNA was extracted using QIAGEN kit (QIAGEN^®^ DNA mini columns kit, QIAGEN, Germany) as instructed by the manufacturer. DNA was stored at −20°C for PCR amplification of targeted ICP4 gene. Following the isolation of the virus in chicken embryonated eggs, further DNA extraction and molecular detection were carried out.

#### 2.5.3 Master mix preparation

Conventional PCR was performed according to the method described by (22) using a pair of forward primers, ICP4-1F (5′-ACTGATAGCTTTTCGTACAGCACG-3′) and reverse primer, ICP4-1R (5′ CATCGGGACATTCTCCAGGTAGCA-3′) that amplify 688 bp fragments of the ILTV ICP4 gene (GenBank accession number: NC_006623) (23). The 18.5 μL amplification reaction containing 3 μL of RNase free water, 5 μL of dNTP mix (2 mM each of dATP, dCTP, dGTP and dTTP), 2 μL each of the primers, 1.5 μL (2.5 units) Dream Taq^TM^ DNA polymerase, 5 μL of 10 × Taq DNA polymerase buffer was mixed with 5 μl extracted DNA to run the PCR.

#### 2.5.4 PCR reaction

The following PCR condition was applied: initial denaturation at 94°C for 3 min, followed by 35 cycles of 94°C for 15 s (denaturation), 60°C for 45 s (annealing) and 72°C for 150 s (extension), and for final extension incubated at 72°C for 3 min. In each series of PCR amplification a sterile distilled water, instead of extracted DNA, was included as a negative control.

PCR products were visualized by agarose gel (1.5%) electrophoresis using PRONASAFE dye. Loading dye (4 μL) was added into 20 μL PCR product, then 10 μL of the mixtures was loaded into each well. A 10 μL of molecular ladder (GelPilot DNA molecular weight marker, QIAGEN, Germany) starting at 100 bp, negative, and positive controls were added in addition to test sample. Electrophoresis was run for 80 min at 120 V. Then the result is read using UV light. The expected product is about 688 bp for ILTV ICP4 gene (22).

### 2.6 Data analysis

Data generated from the study were arranged, coded, and entered into an Excel spreadsheet (Microsoft^®^ excel 2016, Microsoft Corporate, USA). The data were checked for errors of entry and then imported to STATA16 (Stata Corp., College Station, TX, USA) for descriptive and further statistical analyses, Pearson's Chi-square was used to analyze differences in seroprevalence and assess association between risk factors and ILT infection. Statistical significance was determined at *p* < 0.05.

## 3 Results

### 3.1 Seroprevalence

The overall prevalence of ILTV in the four districts of Central Gondar Zone (Gondar, Maksegnit, West dembya, and East Dembya) was 19.4% (Table 3). The highest seroprevalence recorded was 31.6% in the West Dembya area, while the lowest was 12.2% in the Maksegnit area, with significant differences (*p* < 0.05). The mean antibody titer was 764.5 ± 1920.63. The highest mean antibody titers for Infectious Laryngotracheitis (ILT) in backyard chickens were recorded at 990.19, while commercial chickens had a highest titer of 426.0168. Overall seroprevalence was 22.9% (53 out of 231) in backyard poultry systems and 14.2% (22 out of 154) in commercial poultry production systems. Chi square analysis revealed the prevalence was significantly different (*p* < 0.05) between commercial and backyard production systems (Table 4). Local breeds had higher seropositivity rates compared to commercial chickens. Layers were significantly more seropositive (*p* < 0.05) compared to broilers and dual-purpose chickens. On the other hand, the seroprevalence was 16.2% and 20.9% for males and females, respectively. However, there were no significant differences in prevalence based on age and sex (Table 5).

**Table 3:** Seroprevalence of ILTV in four districts in Central Gondar Zone, Ethiopia.

**Location**	**No. samples**	**No positive**	**No negative**	**Prevalence (%)**	***P*-value**
East Dembya	108	23	85	21.2%	0.024
Gondar City	119	21	98	17.6%	
Maksegnit	98	12	86	12.2%	
West Dembya	60	19	41	31.6%	
Total/weighted seroprevalence	385	75	310	21.4%	

**Table 4:** Mean infectious laryngotracheitis virus antibody titers (ELISA) in backyard and commercial chickens in Central Gondar Zone, Ethiopia.

**Variable**	**No**	**Mean Ab**	**Std. Dev**.	**Min**	**Max**	**Prevalence (%)**	***P*-value**
Backyard	231	990.1977	2243.081	1	12388.87	53 (22.9)	0.036
Commercial	154	426.0168	1225.743	1	12,321	22 (14.2)	
Total/weighted seroprevalence	385	764.5254	1920.632	1	12388.87	75 (20.3)	

**Table 5:** Pearson's Chi-square analysis of ILTV seropositivity with various risk factors.

**Variables**	**Level of variable**	**No. sampled**	**No. positive % (number positive)**	**P-value**
Breed	Exotic	241	15.7% (38)	0.017
	Local	144	25.6% (37)	
	Total	385	20.6% (75)	
Sex	Male	123	16.2% (20)	0.274
	Female	262	20.9% (55)	
	Total	385	19.6% (75)	
Age	≤ 6 months	197	15.7% (31)	0.058
	>6 months	188	23.4% (44)	
	Total	385	20.2% (75)	
Purpose	Broiler	166	13.8% (23)	0.006
	Layer	148	27.7% (41)	
	Dual	77	8.4% (11)	
	Total	385	20.6% (75)	

### 3.2 ILTV detection by polymerase chain reaction

Out of 27 oropharyngeal samples, four samples (14.8) were found to be positive for ILT virus ICP4 gene, in which two samples were collected from West Dembya (2/8, 25%), one sample from Gondar city (1/6, 16.6%) and one sample was from East Dembya (1/7, 14.2%) (Table 6). Three of the positive oropharyngeal swabs were from backyard chickens (3/13, 23.4%), while one sample was from commercial chicken farms (1/14, 7.1%) (Table 7). Similarly, three positive samples were detected in broiler chickens (3/7, 42%), and one was from layer chickens (1/13, 7%) (Table 8).

**Table 6:** Molecular detection of infectious laryngotracheitis virus in four districts in Central Gondar Zone, Ethiopia.

**Location**	**No. samples**	**No. negative**	**No. positive % (number positive)**
East Dembya	7	6	14.2% (1)
Gondar City	6	5	16.6% (1)
Maksegnit	6	6	0
West Dembya	8	6	25% (2)
Total	27	23	4

**Table 7:** Molecular detection of infectious laryngotracheitis in backyard and commercial chicken.

**Type of chicken**	**No. of samples**	**No. negative**	**No. positive(%)**
Backyards	13	10	3 (23%)
Commercial	14	13	1 (7.1%)
Total	27	23	4

**Table 8:** Molecular detection of infectious laryngotracheitis in backyard and commercial chicken.

**Chicken purpose**	**No. of samples**	**No. negative**	**No. positive % (number positive)**
Broiler	7	4	42% (3)
Layer	13	12	7% (1)
Dual	7	7	0% (0)
Total	27	23	4

**Figure 1:**
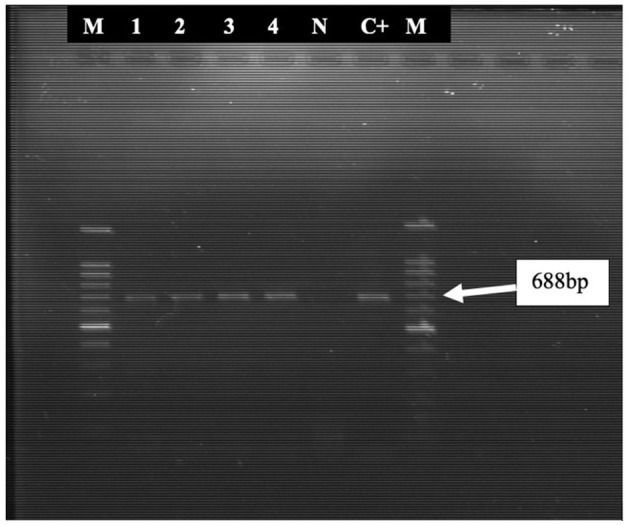
PCR amplified products (688 bp fragments) of the ICP4 gene of ILTV. Lane 1, 2, 3, and 4 were positive for the 688-bp fragment. M = 100-bp DNA ladder; N, Negative control; C+, Positive control (ILTV reference strain).

Initially, only 3 samples (pre-inoculated sample suspension) tested positive using PCR, but the detection rate increased to 4 following culture in embryonated chicken egg. These four oropharyngeal swab samples were positive for ICP4 gene of ILTV. The positive band also appeared in the reference strain used as positive control while no band was detected in the negative control (Figure 1).

## 4 Discussion

In total, 385 samples were collected and tested against ILTV using an indirect ELISA test. An overall seroprevalence of 19.4% was obtained. A positive serology result indicates that ILTV is circulating in Amhara Region since there is no vaccination against ILTV provided. Ethiopia has adopted a non-vaccination policy against ILTV (10). Thus, the findings of this investigation point to either a previous or active ILT infection throughout poultry facilities across a number of districts. It might result from ILTV infection of the field type or from the latent virus reactivating in birds that have recovered from previous infection. ILTVis latent in the trigeminal ganglia of recovered birds until its reactivation by stress, which leads to its excretion and further lateral spread (24).

The first report of Infectious Laryngotracheitis was published in 2019, in central and south Ethiopia based on serological tests. The overall prevalence was higher in backyard poultry (34.4%) than commercial poultry production (13.3%) (10). This was followed by a report in which the highest percentage of prevalence 54.7% in Ada'a district of Bishoftu city in Ethiopia (16). The finding is consistent with reports from (25), Baksi et al. (26), and Langeroudi et al. (14) who demonstrated overall seroprevalence of 17.33% in Chittagong district in Bangladesh, 26.77% in India, and 13% in Iran respectively. However, the seroprevalence found in this study was lower than a previous report of 59.1% from the same region (17) and others' around the globe (11, 27). Conversely, our findings are higher than those reported by Bhuiyan et al. (28) (0.4%), Ana et al. (29) (0.194%), and Shittu et al. (30) (1.2%). Variations in seroprevalences can be attributed to differences in chicken breed, flock age, flock density, biosecurity practice, vaccination status, and test sensitivity and specificity used (31, 32). In this study, seropositivity of ILTV infection was higher in backyard poultry (22.9%) than commercial poultry production (14.2%). The lack of biosecurity measures observed during sample collection from backyard poultry may have increased the risk of exposure in these chickens, potentially contributing to the observed difference. Several other studies support this finding. Several other studies have corroborated this finding (7, 10, 11). Also breed effects on seroprevalence were observed for ILTV. The higher seropositivity of local chickens for ILTV compared to exotic chickens may be associated with local breeds being able to survive better than exotic breeds after ILTV infection.

Up to now, no studies had been conducted on the molecular detection of ILTV in Amhara region. This is the first documentation of ILT infection in Amhara region using PCR. But the first molecular evidence of ILTV in Ethiopia came from a study that confirmed the viral genome from two diseased chickens as part of an investigation of respiratory pathogens of chickens in Central Ethiopia (7). In the present study, clinical signs observed include respiratory signs such as mucoid to hemorrhagic nasal discharge, dyspnea, conjunctivitis, lacrimation, a high rate of morbidity, and low rate of mortality. Our observations agree with those reported by Salhis et al. (11), Ponnusamy et al. (33), Gowthaman et al. (31), García and Spatz (34), and Menendez et al. (35).

Although clinical findings observed during sample collection can help in diagnosing suspected ILT cases, laboratory confirmation is essential (24). Hence, confirming a mild infection of ILT requires laboratory tests that detect the presence of the virus (29). PCR is a method of choice to confirm ILTV infection in chickens with clinical signs suggestive of ILT disease (36). In the present study, the detection of the virus from the oropharyngeal swabs by PCR confirmed the results.

According to PCR results, three swab samples showed positive results out of 27 samples that were collected. To confirm the result, all the samples were inoculated into the CAM of embryonated chicken eggs, followed by virus detection in four samples by PCR. This suggests that the load of the virus in some samples was very low before isolation in embryonated chicken eggs. The positive results by ELISA test and PCR confirmation, which were consistent with ILT's characteristic symptoms that were observed during sample collection, showed that ILTV is circulating in both commercial and backyard chicken, with synchronous higher seroprevalence in Ethiopian backyard chicken. The primer in this study targeted the ICP4 gene of ILTV. The ICP4 is an ~4,386 nucleotide transcriptional protein that regulates ILTV gene expression (37), specifically involved in regulation of gene expression early in infection and is commonly used in epidemiologic studies for characterization of circulating virus strains (38). The gene could serve as a good target for RFLP analysis (22). The PCR results obtained in this study were in agreement with previous reports using the ICP4 gene (39) including Fagbohun et al. (40) in Nigeria, and Rojs et al. (23) in Slovenia. The findings on backyard chickens in this study are unbiased, as samples were taken from unvaccinated chickens, eliminating false-positive results despite rumors of commercial chicken vaccination (34).

Although the vaccine is not officially permitted to be administered in Ethiopia, there is ongoing debate on its use due to rumors that some commercial poultry farms were vaccinating their flocks against the disease (16). Several recent studies have confirmed that partial sequencing and analysis of the ICP4 gene can successfully differentiate between vaccine and field ILT strains (35). PCR-RFLP, sequencing of PCR products for strain differentiation is regionally dependent. Partial sequencing of the ICP4 has been used successfully in both Africa and the Middle East (35). The prevention, control, and source of ILTV infection in chicken might be better understood with additional studies using large sample size and molecular methods.

The lack of hygiene and biosecurity measures that were seen during sample collection in local chickens may be the reason that infections are more prevalent in the local chickens than in commercial flocks. Also the ability of ILTV to persist in a latent form beyond recovery and to reactivate when the host is under stress highlights the significance of avoiding co-infections to reduce host stress. Co-infection treatment is vital to ILT management and should not be neglected (37).

However, mixed respiratory infections are challenging to identify because typically only one pathogen is required for a diagnosis (11). An essential component of ILT management is co-infection prevention. Additionally, it is important to consider the possibility of pathogen exposure and transfer from small flocks to commercial flocks.

Ana Garrido et al. (29) observed that all birds are susceptible to infection, but clinical disease affects layers more frequently. Our findings support this observation because laying chickens made up the majority of positive farms. They may have a longer productive life than broilers, which could account for higher infection. On the other hand, protected flocks must have a high average antibody titer from the baseline arising from vaccination, with no specific clinical indications (11). In the present study, the differences in the seroprevalence of ILT among districts were statistically significant.

ILTV strains can spread from flocks that have been continuously infected to birds that are not vaccinated (36). Hence, the authors suggest the source of the ILT infection in this investigation was introduced by carrier birds that are reservoirs, and unvaccinated stressed flocks. Modified live vaccines must be used in these situations, even though they can induce latent infection and spread quickly to nearby flocks. It is necessary to establish continuous epidemiological surveillance to evaluate the incidence and prevalence of the disease using conventional methods like isolation in embryonated chicken eggs (ECE), PCR, PCR-RFLP, and sequencing of PCR products. In order to avoid disease and lessen its economic impact, proper biosecurity procedures and good hygiene are essential.

## 5 Conclusions

- The present study confirms the presence of ILTV infection in commercial and backyard chickens in Amhara region, Ethiopia, using serological and molecular methods. The study showed that the backyard and commercial chickens in Amhara region, Ethiopia, shed the virus with the potential of spreading the infection to other birds, owing to low herd immunity to ILTV. The result emphasizes the need to implement a control program of ILT based on vaccination strategy using recombinant viral vector vaccine and standard biosecurity measures in order to avoid further ILT outbreaks and reduce the risk of widespread virus transmission.
- Furthermore, to identify the circulating strains and define their temporal and geographic distribution, particularly among backyard chickens, further extensive research is critically needed.
- The potential virulence of ILTV strains, prevention, control, and the source of ILT infection could all be better understood with additional research on more samples and molecular characterization.

## Data Availability

The original contributions presented in the study are included in the article/Supplementary material, further inquiries can be directed to the corresponding author/s.
