# Supplementary material for: Serological and molecular detection of infectious laryngotracheitis virus in chickens in Central Gondar Zone, Ethiopia
Source: Front Vet Sci. 2025 Mar 3;12:1517373. doi: 10.3389/fvets.2025.1517373 (PMC11913453; doi:10.3389/fvets.2025.1517373)
Supplement: Supplementary file 1 [file Data_Sheet_1.ZIP › analysis result.pdf]

```
save "C:\Users\USER\Desktop\ILT Writinig\Data\stata last.dta", replace
file C:\Users\USER\Desktop\ILT Writinig\Data\stata last.dta saved

. tab breed

     breed |      Freq.     Percent        Cum.
------------+-----------------------------------
   bovance  |        104       27.01       27.01
     local |        144       37.40       64.42
     sauso |        137       35.58      100.00
------------+-----------------------------------
     Total |        385      100.00



. tab ELISAresult breedcode, chi2

ELISAresul |       breed code
         t |         0          1 |     Total
-----------+----------------------+----------
  Negative |       203        107 |       310
  Positive |        38         37 |        75
-----------+----------------------+----------
     Total |       241        144 |       385

          Pearson chi2(1) =   5.6629   Pr = 0.017

. tab ELISAresult Agecode, chi2
```

```
ELISAresul |            Age code
         t |         0          1          6 |     Total
-----------+---------------------------------+----------
  Negative |       165        144          1 |       310
  Positive |        31         44          0 |        75
-----------+---------------------------------+----------
     Total |       196        188          1 |       385

          Pearson chi2(2) =   3.7649   Pr = 0.152
```

. replace Agecode = 0 in 101

(1 real change made)

. save "C:\Users\USER\Desktop\ILT Writinig\Data\stata last.dta", replace

file C:\Users\USER\Desktop\ILT Writinig\Data\stata last.dta saved

. tab ELISAresult Agecode, chi2

```
ELISAresul |       Age code
         t |         0          1 |     Total
-----------+----------------------+----------
  Negative |       166        144 |       310
  Positive |        31         44 |        75
-----------+----------------------+----------
     Total |       197        188 |       385

          Pearson chi2(1) =   3.6062   Pr = 0.058
```

. tabulate ELISAresult sexcode, chi2

ELISAresul t	sex code 0	1	Total
0	103	207	310
1	20	55	75
Total	123	262	385

Pearson chi2(1) = 1.1950 Pr = 0.274

. tabulate ELISAresult Purposecode, chi2

ELISAresul t	Purpose code 0	1	2	Total
0	137	107	66	310
1	23	41	11	75
Total	160	148	77	385

Pearson chi2(2) = 10.3623 Pr = 0.006
