# Supplementary figures and images for: Serological and molecular detection of infectious laryngotracheitis virus in chickens in Central Gondar Zone, Ethiopia
Source: Front Vet Sci. 2025 Mar 3;12:1517373. doi: 10.3389/fvets.2025.1517373 (PMC11913453; doi:10.3389/fvets.2025.1517373)

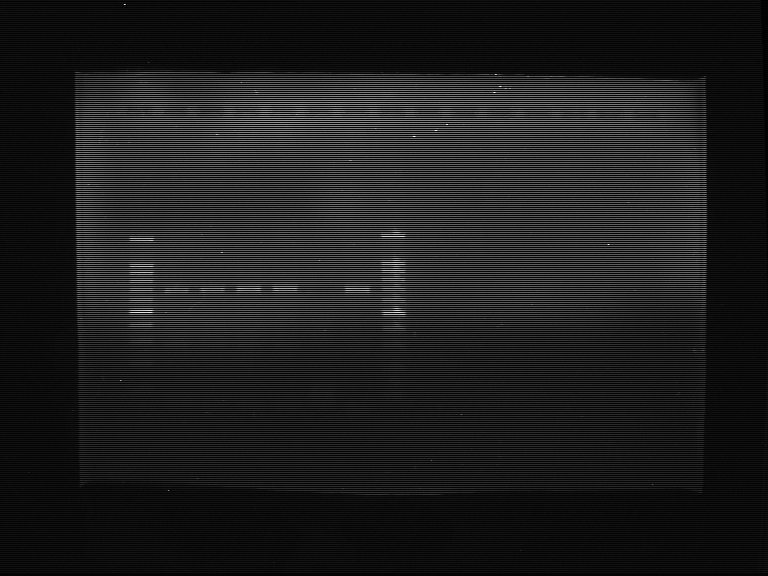

Supplement: Supplementary file 1 [file Data_Sheet_1.ZIP › IM000098.Tif]

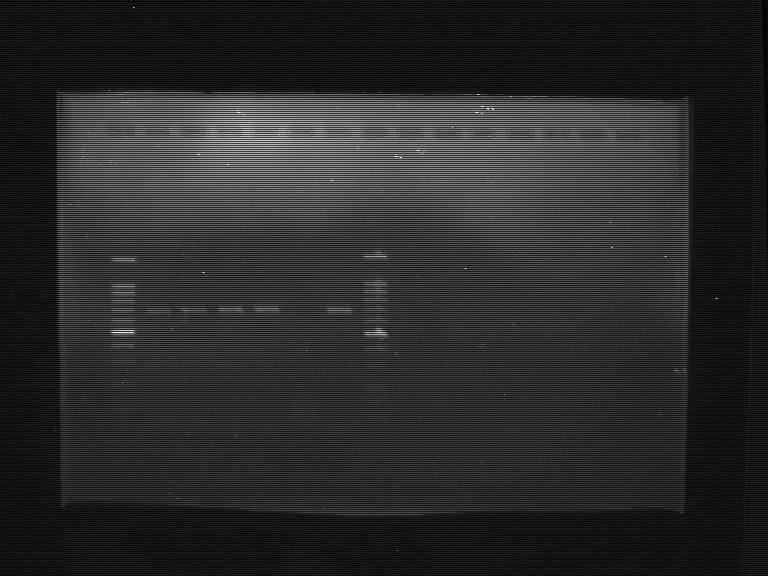

Supplement: Supplementary file 1 [file Data_Sheet_1.ZIP › IM000099.Tif]

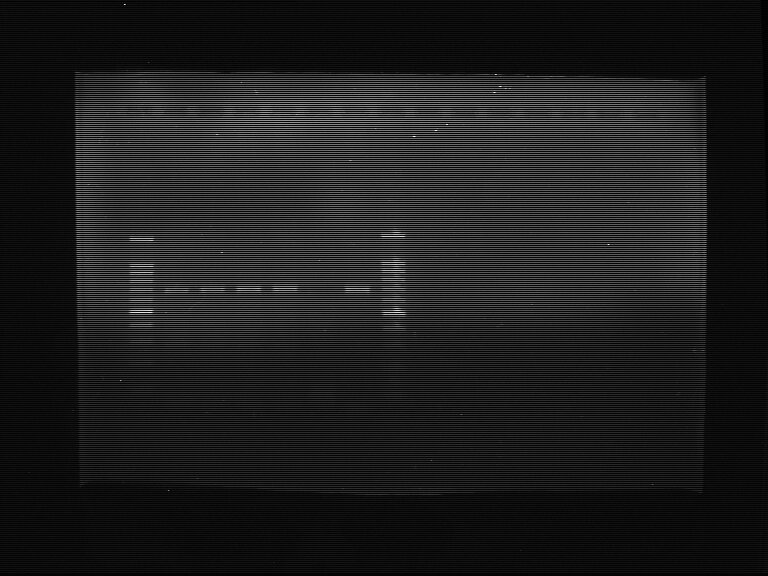

Supplement: Supplementary file 1 [file Data_Sheet_1.ZIP › Pcr 1 ed jpeg.jpg]
